# Identification of a peptide for folate receptor alpha by phage display and its tumor targeting activity in ovary cancer xenograft

**DOI:** 10.1038/s41598-018-26683-z

**Published:** 2018-05-30

**Authors:** Lijun Xing, Yifeng Xu, Keyong Sun, Hong Wang, Fengguo Zhang, Zhengpin Zhou, Juan Zhang, Fang Zhang, Bilgen Caliskan, Zheng Qiu, Min Wang

**Affiliations:** 1 School of Life Science and Technology, China Pharmaceutical University, Nanjing, 210009 P.R. China; 2 Jiangsu Collaborative Innovation Center of Chinese Medicinal Resources Industrialization, School of Pharmacy, Nanjing University of Chinese Medicine, Nanjing, 210023 P.R. China

## Abstract

The expression level of folate receptor alpha (FRα) is located highly rate in ovarian cancer though it is remained absent in normal tissues. This highly tumor restricted expression profile makes FRα a promising target for tumor therapy and diagnosis. In this research we report a FRα binding peptide C7(Met-His-Thr-Ala-Pro-Gly-Trp-Gly-Tyr-Arg-Leu-Ser) discovered by phage display and this peptide showed specific binding to FRα expressing cells by cell ELISA and flow cytometry. Tumor targeting ability of C7 was proved *in vivo* by both phage homing experiment and fluorescence imaging. C7 can be internalized by SKOV3 cells and its affinity to FRα was determined by MST. The molecular recognition was revealed by structure modeling, suggesting its binding mode with FRα.

## Introduction

Folate receptor alpha (FRα) is a 38-kDa glycosylphosphatidylinositol (GPI)-anchored glycoprotein that binds physiological folates and mediates their intracellular transport^1^. It is found that FRα expression in normal tissues is highly restricted to the apical surface of polarized epithelial cells and moreover it is inaccessible to the normal circulation^2^. FRα is significantly up-regulated in a variety of epithelial cancers such as serous and endometrioid epithelial ovarian cancer (OC), endometrial adenocarcinoma, non-small cell lung cancer (NSCLC) of the adenocarcinoma subtype, and to a less extent in clear cell renal, colorectal and breast cancers to meet the folate demand of rapidly dividing cells under low folate conditions^3–5^. In nearly 90% of the non-mucinous ovarian cancer, FRα expression has been observed and correlated with tumor grade, stage, aggressiveness and its expression is maintained after chemotherapy in epithelial ovarian and endometrial cancer^6,7^.

FRα can be exploited for cancer diagnostics and treatments^8^. A range of FRα-targeting approaches, including folic acid derivatives, folate drug-conjugates and small molecules, vaccines, T-cell therapies and monoclonal antibodies (mAbs), have been developed for both imaging and therapeutic purposes^9–12^. Monoclonal antibodies can bind specifically to FRα with high affinity. Although various anti-FRα antibodies have been generated and investigated, the clinical results were not successful. Farletuzumab, a humanized monoclonal antibody targeting FRα, showed a substantial efficacy in both preclinical and Phase I/II clinical studies, whereas no improvement in progression-free survival (PFS) was observed in Phase III clinical trial^13,14^. Furthermore, the large molecule weight of antibody causes difficulties in tumor infiltrating and large scale manufacture. In addition to that, the Fc region of the antibodies binds to the reticuloendothelial system, resulting in significant toxicities to liver, bone marrow, and spleen. Preclinical studies of anti-FRα TS inhibitors including CB300638 and ONX-0801 have demonstrated anti-tumor effect in FRα-expressing tumors^15,16^. However, those inhibitors could reduce folic acid transportation through reduced folate carrier (RFC), which is ubiquitously expressed, leading to non-specific effect which is related with reductions in patient tolerability^15,16^. It is clarified that affinity of folate for FRα is significantly high regarding to the interactions between the receptor and ligand. The folate pteroate moiety is buried inside the receptor, whereas its glutamate moiety is solvent-exposed and sticks out of the pocket entrance^17^. This binding mode allows folate to be conjugated to drugs without adversely affecting FRα binding. Various folate conjugates have been developed for tumor therapy and imaging. However, as summarized by Cheung^8^, folate conjugates have several disadvantages. Firstly, folate conjugates target both FRα and the functional form of FRβ. Unfortunately, it was also possible to target infiltrated tumor-associated macrophages (TAMs), known to express FRβ without selectivity. Secondly, the binding of folate conjugates might be competitively-inhibited by excess serum free folate. Thirdly, folate conjugates could be taken up by RFC, which is expected to confer patient toxicity.

Considerably, the tumor restricted distribution of FRα and its emerging roles in cancer development make it a potential target especially for ovarian cancers. Novel approach has to be developed upon this tumor marker. As drug candidates, synthetic peptides have higher specificity than small molecule drugs. While comparing with antibodies, peptides have reduced immunogenicity, rapid blood clearance, increased diffusion and tissue penetration, chemical stability and ease of synthesis^18^. Widespread use of targeted therapies in the clinic requires high affinity, tumor-specific agents as effective targeting vehicles to deliver therapeutics to the tumor sites. Several peptides have already been used for tumor targeting and treatments. Tumor targeting peptides are efficient vehicles for selective delivery of high dose of chemotherapeutic drugs or diagnostic agents to tumor sites while sparing normal tissues. And till now there are few researches of FRα targeting peptides. Peptide phage display is a powerful method in discovering specific binding sequences with diverse targets like proteins^19^, enzymes^20^, virus^21^, cells^22^, tissues or organs^23^ and provides a wide range of application possibilities in drug discovery. Therefore, we conduct a research in order to discover FRα binding peptides using phage display.

## Results

### Screening and Identification of FRα Binding Peptides

**Figure 1:**
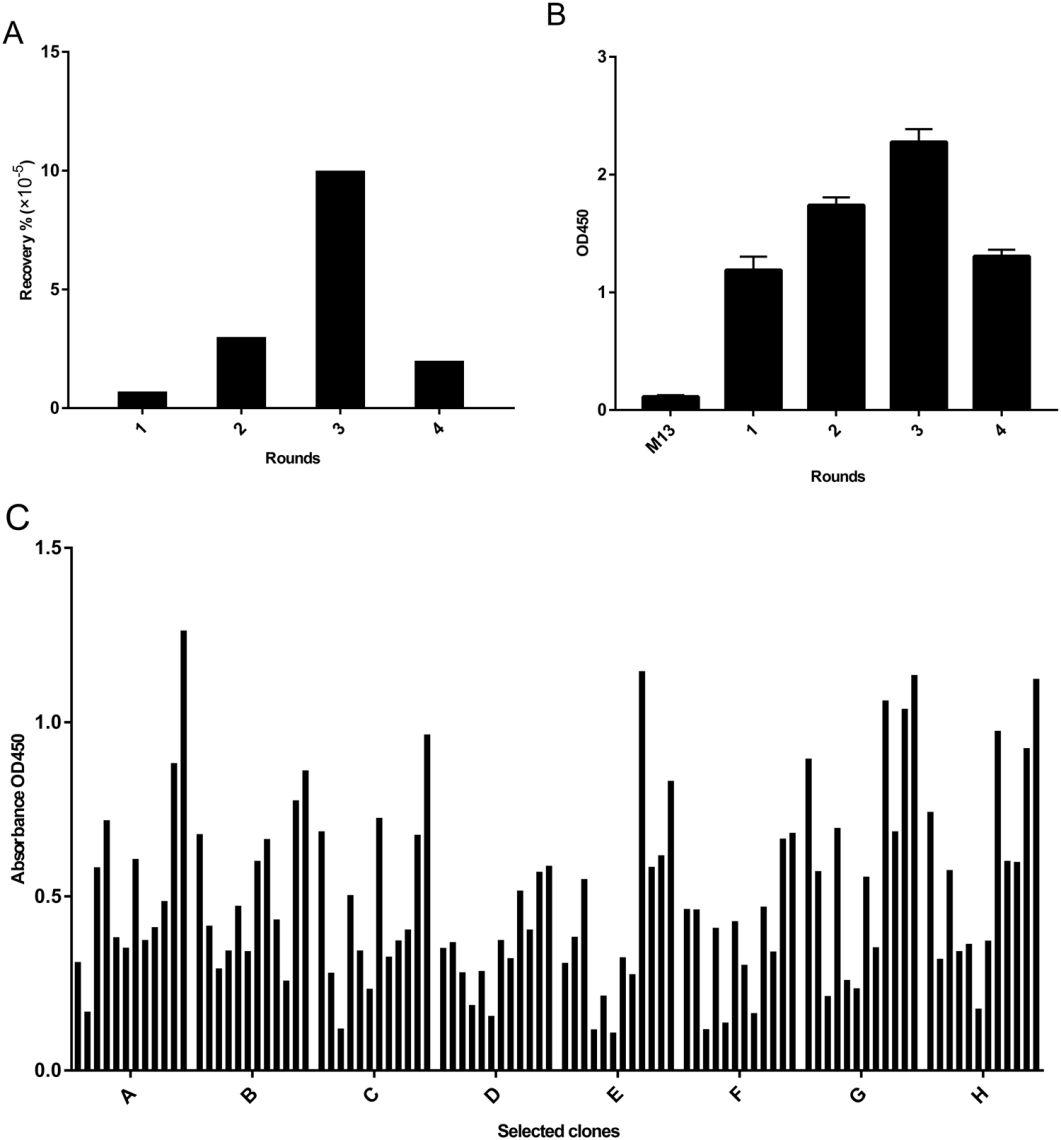
Screening and identification of FRα binding peptides. A Ph.D.-12 phage library was used to screen FRα binding phages with four rounds of biopanning. (**A**) The enrichment of FRα binding phages were evaluated by phage recovery yields of each round selection. (**B**) Polyclonal phage ELISA using elutes after each round selection. (**C**) 94 phage clones were randomLy picked from the third round selection, and their binding affinities for FRα were analyzed individually by phage ELISA.

A Ph.D.-12 phage library displaying random dodecapeptides was used to isolate FRα binding phages with four rounds of biopanning. Phage yields from each round were used to indicate the enrichment of phages. After each round of biopanning, the bound phages were eluted and elutes were tested by phage ELISA to evaluate the affinity to FRα. It was demonstrated by phage yield and polyclonal phage ELISA that the FRα-binding phages increased as the biopanning rounds increased in the first three rounds screening, indicating that the FRα binding phages were enriched effectively in biopanning process (Fig. 1A and B). Phage recovery and ELISA value did not increase after the fourth round of screening, suggesting 3 rounds of panning were sufficient for FRα binding phages enrichment. From the third round selection, 94 phage clones were randomLy picked and their binding affinities for FRα were analyzed individually using phage ELISAs. All of the selected phage clones showed higher FRα-binding affinity than did the wild-type M13 clone (Fig. 1C). Among the phage clones analyzed, 20 clones with the highest absorption values were sequenced to determine the amino acid sequences of the displayed peptides (Table 1). Several clones were found identical and totally 15 different clones were identified.

**Table 1:** Peptide sequences of phage clones binding to FRα in random twelve peptide library.

Clones	Peptide Sequence
A3	Leu-Gly-Ile-Ser-Ala-Thr-Asn-Ala-Tyr-Ala-Arg-His
A4	Phe-Ser-Gln-Ala-Thr-Gly-Arg-Ser-Pro-Thr-Thr-Leu
A7	Ala-Ser-Val-Leu-Asp-Tyr-Lys-Gly-Phe-Phe-Gln-Arg
A12	Thr-Ala-Ala-Gln-Trp-Phe-Pro-Ser-Leu-Ser-Asn-Asn
B1	Asn-Trp-Gln-Pro-Thr-Ala-Gly-Leu-Lys-Pro-Leu-His
B7	Trp-Ser-Ala-Ala-Thr-Val-Pro-Arg-Gly-Phe-His-Ala
B8/H1/G4	Gly-Ala-Leu-Leu-Pro-Ser-Met-Asn-Lys-Gly-His-Trp
C1	Ser-Asn-Ser-Asp-Ala-Tyr-Ala-Leu-Gln-Phe-Leu-Arg
C7	Met-His-Thr-Ala-Pro-Gly-Trp-Gly-Tyr-Arg-Leu-Ser
E9	Phe-Phe-Pro-Leu-Thr-Phe-Pro-Trp-Thr-Tyr-Tyr-Asp
G1/G10	Ser-Gly-Val-Tyr-Lys-Val-Ala-Tyr-Asp-Trp-Gln-His
G9, D5	Val-His-Trp-Asp-Phe-Arg-Gln-Trp-Trp-Gln-Pro-Ser
G8	Tyr-Thr-Asn-Pro-Tyr-Tyr-Ser-Ser-His-Thr-Arg-Asn
G12	Met-Asn-Pro-Tyr-Pro-Arg-Thr-Pro-Trp-Pro-His-Val
H11	Arg-Gly-Met-Asp-Thr-Leu-Trp-His-His-Ala-Tyr-His

20 clones with the highest ELISA abortion values were sequenced to determine the amino acid sequences of the displayed peptides. 15 clones with individual peptide sequence were identified.

### Binding to FRα Expressing Cells

**Figure 2:**
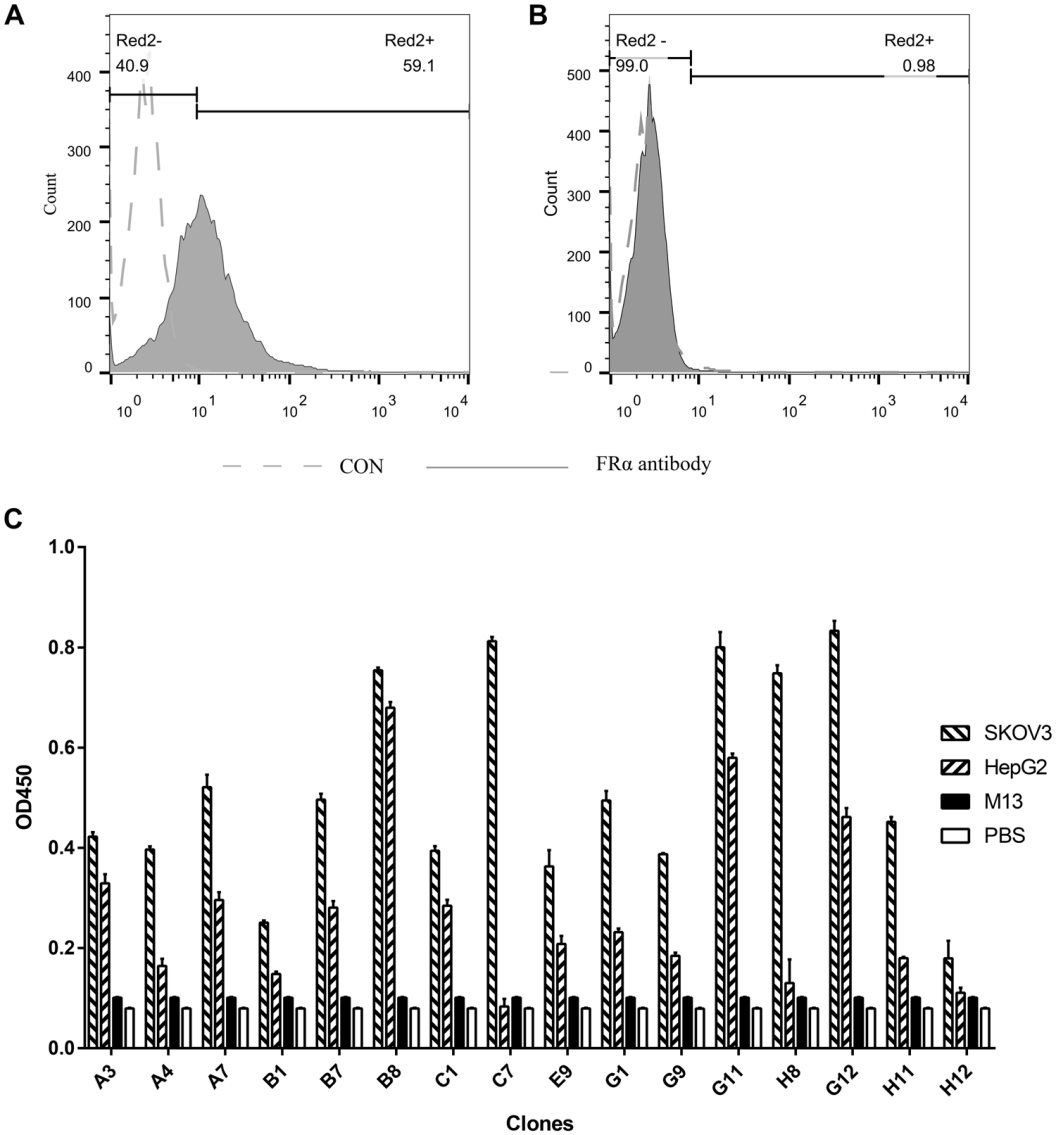
Phage binding to FRα expressing cells. SKOV3 and HEPG2 were tested for their FRα expression by flow cytometry and used as positive and negative cells in cell ELISA. (**A**) The expression of FRα on SKOV3 cells. (**B**) The absence of FRα expression on HEPG2 cells. (**C**) Phage clones were further screened by cell ELISA.

**Figure 3:**
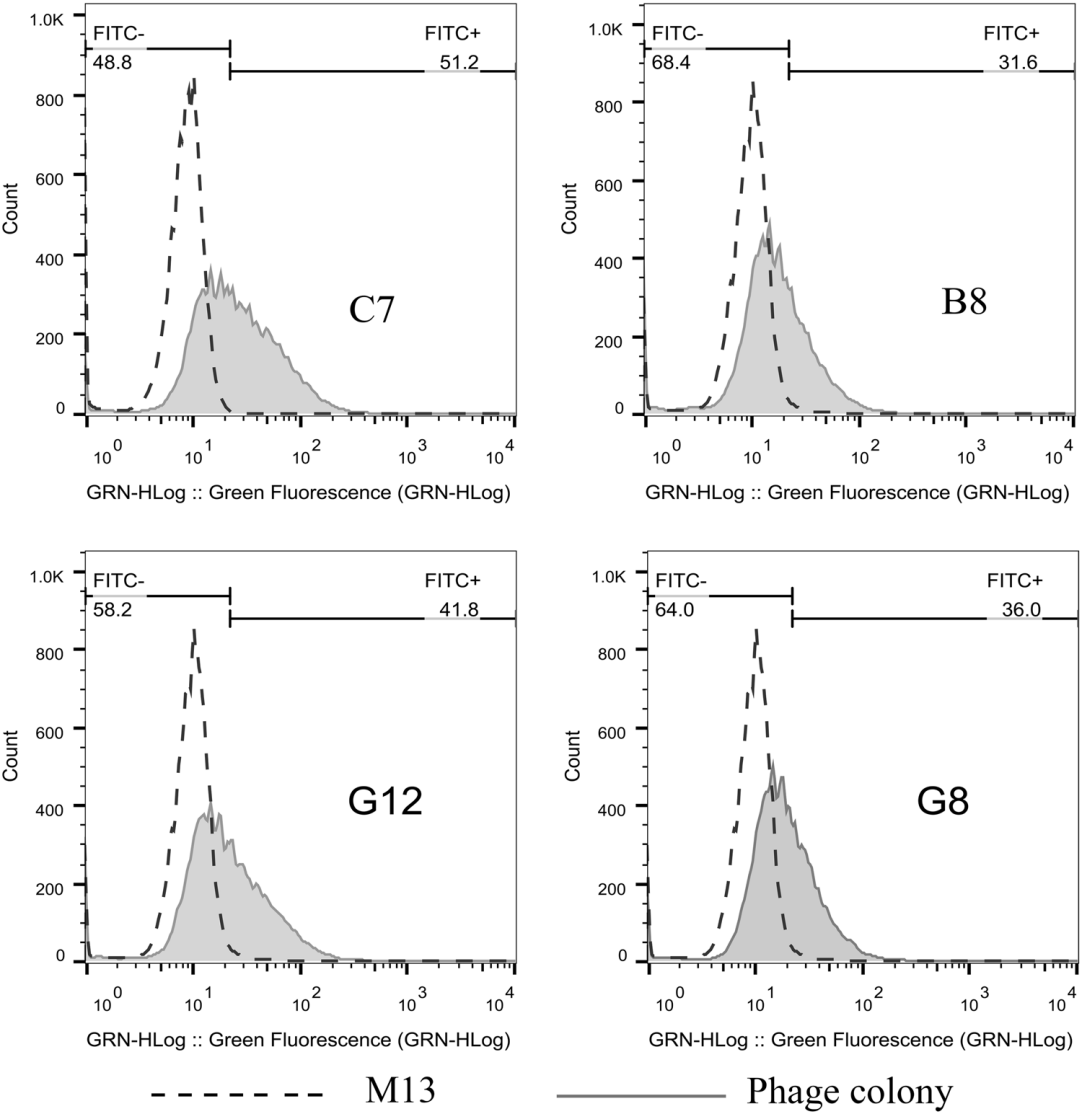
Phage binding to SKOV3 cells was confirmed by flow cytometry. Four phage clones (C7, B8, G12, G8) revealed specific binding (51.2%, 31.8%, 41.8% and 36.0%) to SKOV3 cells. Phage M13 was assessed as negative control.

Since prokaryotic recombinant human FRα protein were used for screening, the 15 selected phage clones were further tested for their binding ability to native human FRα expressed on the mammal cell surface. Firstly, the presence of FRα expression on SKOV3 cells (human epithelial ovarian cancer cell) and the absence of FRα expression on HepG2 cells (human liver hepatocellular carcinoma Cells) were confirmed by flow cytometry (Fig. 2A and B). SKOV3 cells were used as positive cells and HepG2 cells were used as negative cells in the following experiments. Cell-based ELISA was used to evaluate preliminarily the binding properties of phage clones. As shown in Fig. 2C, most selected phages had a higher binding to SKOV3 cells comparing with the binding to HepG2 cells. M13KE, as the negative control, did not bind to either of the cell. 4 phage clones (C7, B8, G12, G8) with the highest binding to SKOV3 and relatively low binding to HepG2 in cell-base ELISA were selected and further evaluated by flow cytometry. In contrast to M13KE, these four clones revealed specific binding (51.2%, 31.8%, 41.8% and 36.0%) to SKOV3 cells (Fig. 3).

### C7 Phage Clone Homes SKOV3 Implanted Tumor *in Vivo*

**Figure 4:**
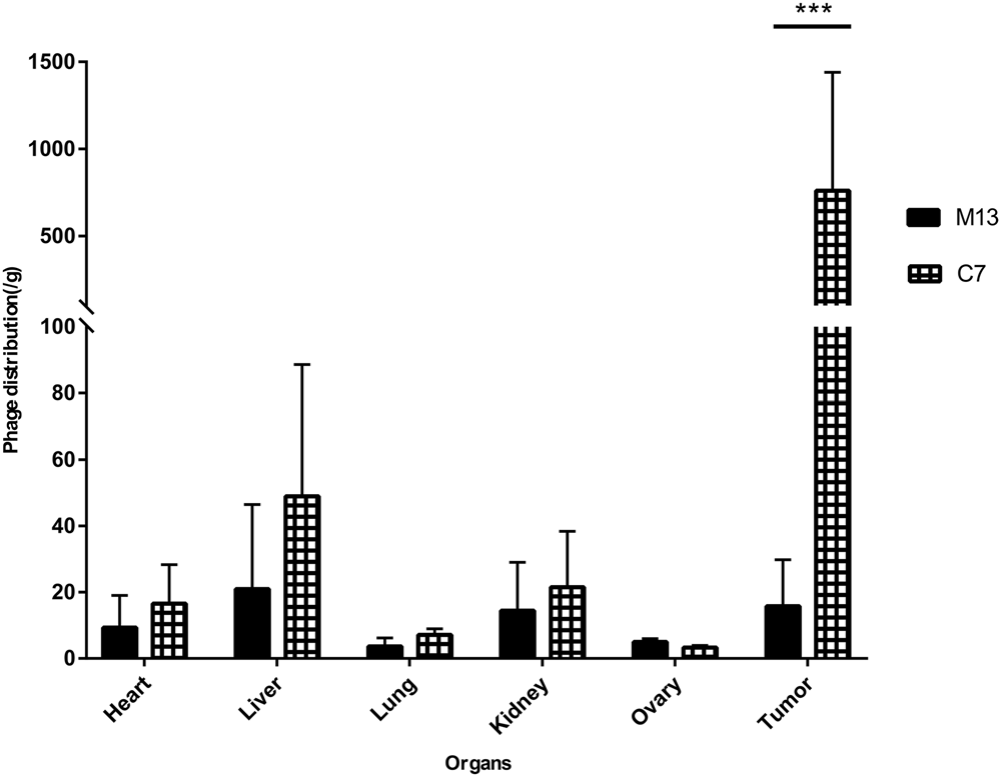
*In vivo* homing of the selected phages in SKOV3 xenografts. Selected phage clones C7, or M13KE phage were amplified and 2 × 10^11^ individual purified phage clones were injected intravenously to tumor bearing mice. The distribution of phage in tumor and normal organ tissues was determined by titration. Results were expressed as titrated phages per gram of tissue. Data were given as the mean ± SD (n = 3).

To test the tumor homing efficacy, 2 × 10^6^ SKOV3 cells were subcutaneously injected into the left flank of Balb/c nude mice to generate epithelial ovarian cancer xenografts. When the tumors reached about 200 mm^3^, the control phage M13KE or purified phage clone (C7) with superior binding ability to SKOV3 cells were injected intravenously and circulated for 15 min. After perfusion, the tumor and main organs were harvested and bound phages were recovered and quantified by phage titration. As shown in Fig. 4, C7 showed significant enrichment in tumor tissue, which was 49-fold more than the M13KE phage in tumor. And the phage titer of C7 in tumor was 190-fold higher than which in the normal ovary. Same amount of phages (2 × 10^11^) were used in different groups and the capsid proteins were the same among different phage clones. Therefore, it was the displayed peptide that resulted in different affinities to tumors. According to the homing test, C7 processes tumor targeting ability *in vivo*.

### C7 peptide binds to SKOV3 cells through FRα

**Figure 5:**
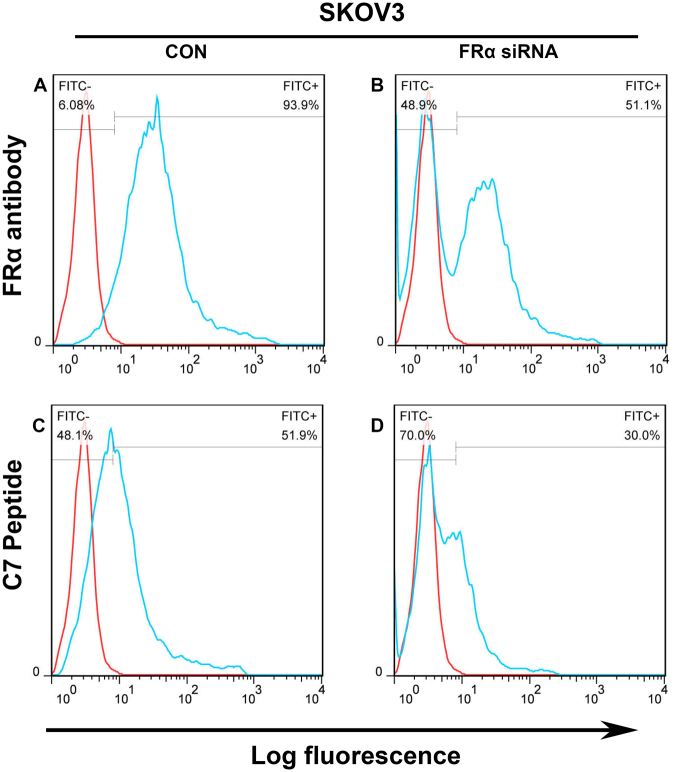
RNA interference diminished the binding rate of C7 peptide to SKOV3 cells. SKOV3 cells were transfected with FRα specific siRNA or control siRNA. After 72 h, siRNA mediated silencing of FRα was tested with monoclonal anti-FRα antibody. It was shown that the expression of FRα was decreased from 93.9% to 51.1%. The binding of C7 peptide to SKOV3 was reduced from 51.9% to 30% after FRα knockdown.

To determine whether C7 peptide targets FRα on SKOV3 membrane, we employed RNA interference to knockdown FRα in SKOV3 cells. SKOV3 cells were transfected with FRα specific siRNA and the silencing effect was confirmed with anti-FRα antibody using flow cytometry. It was observed that expression of FRα was greatly diminished in SKOV3 cells after FRα specific RNA interference (Fig. 5A,B). FITC conjugated peptide C7 was synthesized and tested for its binding to SKOV3 cells and the effect of RNA interference on its bind ability. C7 peptide showed a binding of 51.9% to SKOV3 cells and it was decreased significantly to 30% by FRα specific RNA interference, indicating C7 peptide binds to SKOV3 cells through FRα (Fig. 5C,D).

### Tumor Targeting of Synthesized Peptide by Fluorescence Imaging

**Figure 6:**
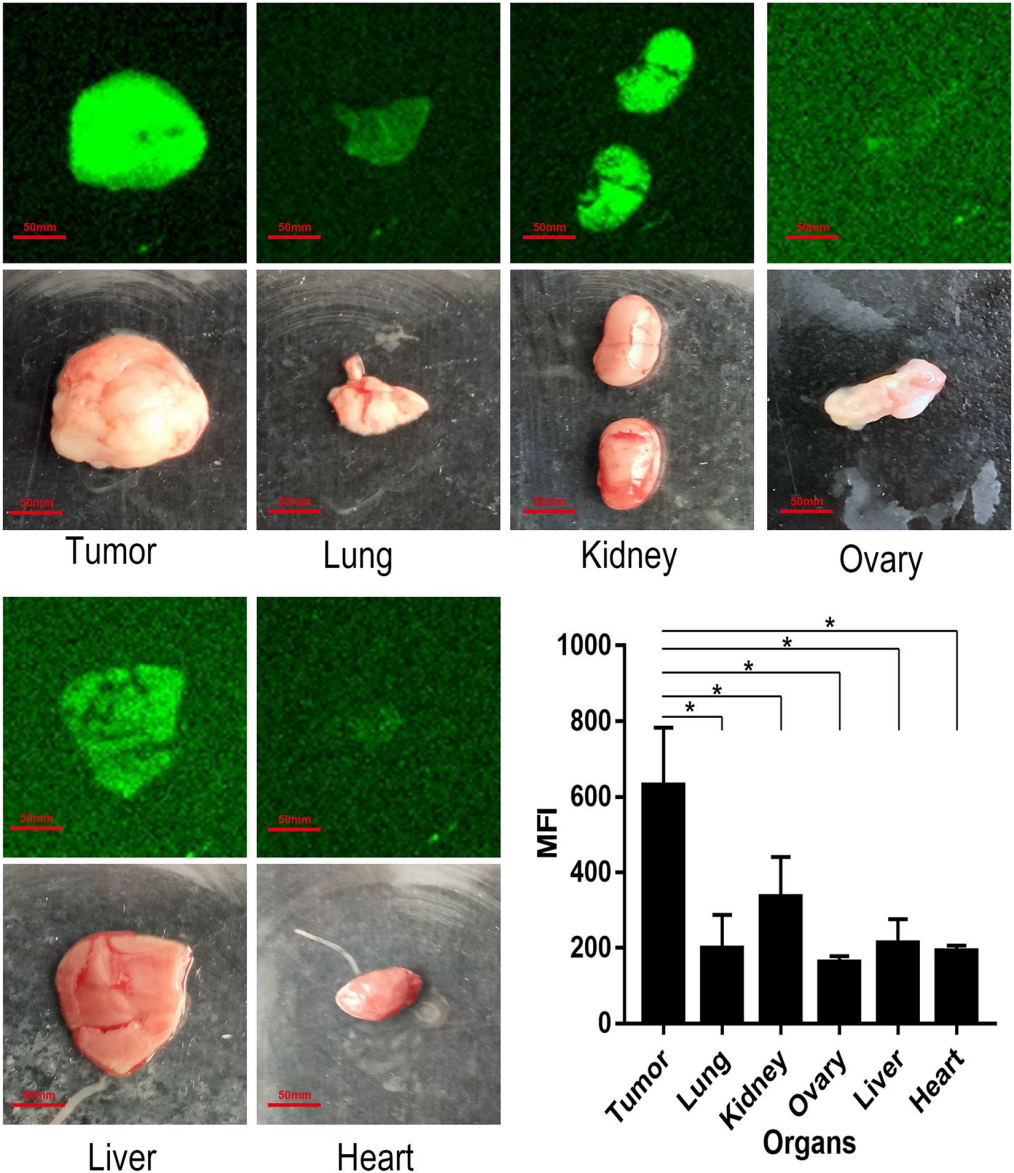
The tumor-targeting efficacy of synthetic peptides *in vivo*. FITC conjugated peptide C7 was chemically synthesized with more than 95% purity. The peptide was dissolved in PBS and injected intravenously into a tumor bearing nude mouse (200 μL, 10 mg/mL). After 2 hours circulation, the fluorescence distributions in tumor and other organ tissues were analyzed with Carestream IS4000 fluorescence imaging system. Data were given as the mean ± SD (n = 3).

FITC conjugated peptide was injected intravenously into a tumor-bearing nude mouse to assess whether the C7 peptide retained tumor targeting capability *in vivo*. After 2 h circulation, fluorescence imaging was used to detect MFI (mean fluorescence intensity) of FITC in tumor and other organ tissues. As shown in Fig. 6, the fluorescence signal observed in the tumor is significantly stronger compared to that in other organs except liver and kidney. MFI of C7 in tumor was detected to be 3.3 times stronger than that in ovary. Thus, it has been demonstrated that the synthesized peptide C7 maintains tumor targeting activity *in vivo*.

### Peptide has High Affinity for FRα

**Figure 7:**
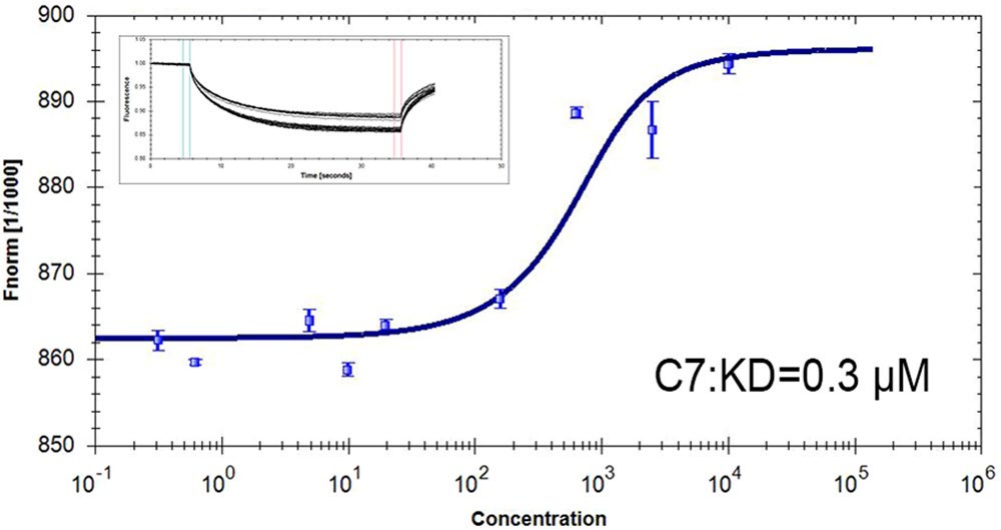
Affinity test. FITC conjugated peptide C7 was diluted with PBS. Serial dilutions of peptide were mixed with purified FRα protein (1 μg/mL) and incubated for 5 min at room temperature. Each sample was loaded into the standard monolith NT capillary and detected in Monolith NT.115. The error bars reflect standard deviation (SD) from 3 measurements.

MST Analysis was performed in furtherance of evaluating binding affinity of C7 for FRα. According to the changes of fluorescent thermophoresis signal caused by the serial dilutions of labeled peptides, the equilibrium dissociation constant (KD) value was calculated from Hill formula by the NT analysis. It was showed that the equilibrium dissociation constant (KD) value between C7 and FRα was 0.3 μM (Fig. 7).

### Cell Internalization of Synthesized Peptide

**Figure 8:**
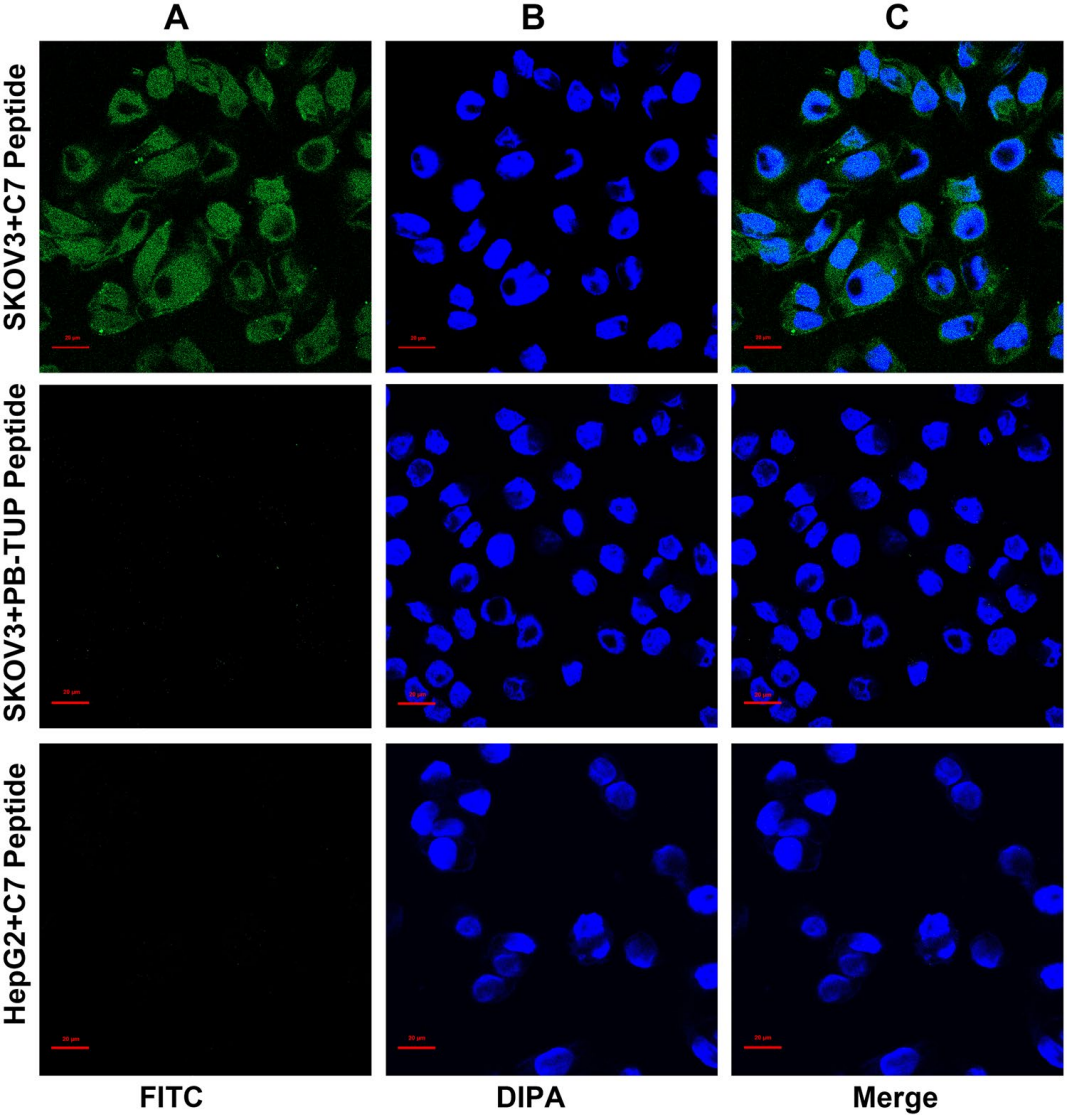
Cell internalization. FITC conjugated peptide C7 or was incubated with SKOV3 cells for 4 hours at 37 °C. Then the cells were washed, fixed and stained with DAPI and viewed by confocal microscopy. (**A**) FITC conjugated C7. (**B**) DAPI stained SKOV3 nuclei. (**C**) Overlay of A and B showing that the C7 is internalized.

FITC conjugated peptide C7 or FITC conjugated irrelevant peptide (PB-TUP) was incubated with SKOV3 cells for 4 hours at 37 °C. After fixation, confocal microscopy was used to visualize the internalization of peptide into SKOV3 cells. In addition, HepG2 cells were used to evaluate the internalization of peptide C7 into FRα low expressing cells. As shown in Fig. 8, the florescence can be observed in the cytoplasm and at the perinuclear region, showing the peptide C7 can be internalized into SKOV3 cells. In contrast, irrelevant peptide was not internalized by SKOV3 cells, and C7 peptide was not detected in HepG2 cells.

### Circular Dichroism Spectroscopy Analysis

**Figure 9:**
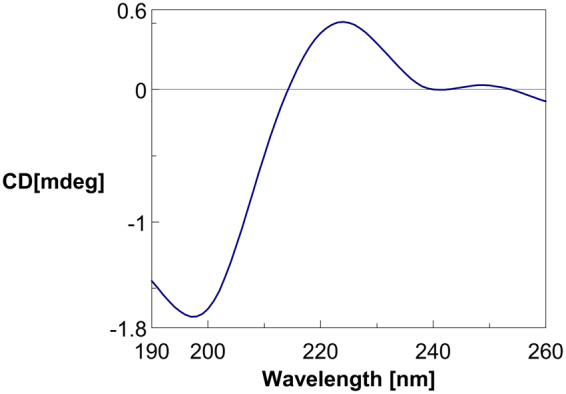
Circular dichroism spectrum of the synthesized peptide C7. The peptide solution (0.3 mg/mL) was prepared using ultra water. Spectra were collected every 1 nm from 260 to 190 nm. The CD spectra are recorded as CD (mdeg).

**Figure 10:**
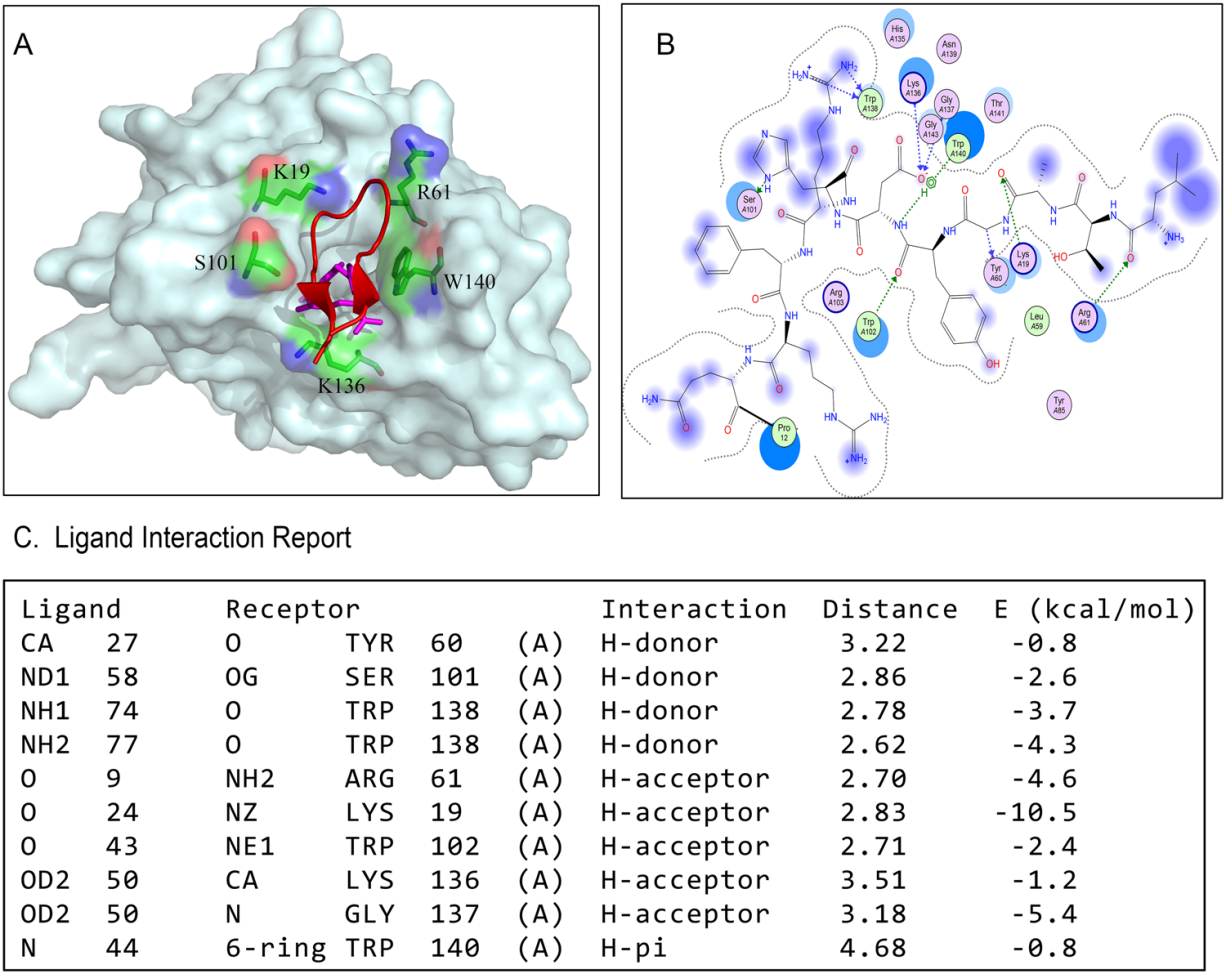
Binding mode of peptide to FRα by molecular docking. (**A**) Overlay of the crystal structures of C7-FRα complexes and folate-FR complexes (4LRH). (**B**) 2D interaction of C7 and FRα. (**C**) Ligand interactions report.

To analyze the secondary structure of the peptide C7, we performed CD spectroscopy. The CD spectrum of C7 exhibited a minimum at 198 nm and a maximum at 220 nm, demonstrating a random coil structure of the peptide (Fig. 9). The results of molecular modeling supported the formation of random coil structure (Fig. 10A). Peptide secondary structures are key determinants of molecular interactions. Random coil structure makes the peptide highly flexible and this structure might help the peptide optimize the arrangement of amino acid residues in binding with FRα.

### Computational Modeling and Interaction Analysis

The 3D structure of C7 was predicted by Pepfold3. As shown in Fig. 10A, C7 forms a random coil configuration. To illuminate the binding mode of peptides, peptide C7 and FRα protein were uploaded to ClusPro server for molecular docking respectively. The most favorable models from the 29 server retrieved complexes were chosen for further research. The interactions between FRα and peptide from the molecular docking were analyzed by MOE. The peptide-FRα complex and folate-FRα complex (PDB ID: 4LRH) were overlapped respectively. FRα has a globular structure stabilized by eight disulfide bonds and contains a deep open folate-binding pocket. Peptide C7, because of its size, cannot stick into the pocket, but binds with FRα close at the entrance of the pocket. C7 forms hydrogen bonds with Tyr60, Ser101, and Trp138 as a H-donor and as a H-acceptor forms five hydrogen bonds with Lys19, Arg61, Trp102, Lys136, and Gly137 (Fig. 10B). In addition, C7 can make a H-π interaction with Trp140.

## Discussion

In this manuscript, we conduct a research to discover tumor targeting peptides using phage display. We performed 4 rounds of screening. In the first 3 rounds, the phage yield and polyclonal ELISA signal both increased, indicating the enrichment of FRα binding phage. However, in the fourth round of selection, the yield and polyclonal ELISA value decreased, suggesting that the eluted pool of phage was fully enriched in favor of binding sequences after 3 rounds. Once this point is reached, further rounds of amplification and panning would result only in selection of phages that have a growth advantage over the library phages. Therefore, individual clones were picked from the third round biopanning for further studied. Interestingly, among the selected peptide sequence, Met-His-Thr-Ala-Pro-Gly-Trp-Gly-Tyr-Arg-Leu-Ser has been isolated repeatedly in our laboratory on different targets and was identified as a polystyrene binding peptide^24^.

Taking into consideration of the difference between the prokaryotic expressed FRα protein and eukaryotic mammal cell surface FRα, the 15 different peptide sequences were screened further by a cell based ELISA. Predicatively, not all the clones bind to SKOV3 cells as well as to FRα protein. Also, a few clones were excluded because of their unspecific binding to HepG2 cells without FRα expression. In flow cytometry, phage clone C7 was confirmed its highest binding to SKOV3 cells and was chosen for an ensuing study. In tumor bearing nude mouse model, the selected phage C7 was validated for its tumor targeting ability by *in vivo* homing experiment.

FITC conjugated peptide C7 was synthesized and studied. The fluorescence group might affect the binding ability of the molecule to be conjugated. By computational docking analysis, we found that amino acids at N-terminus of C7 contribute little to the interaction with FRα, while amino acids at the C-terminus form more stable hydrogen bonds with FRα (Fig. 10B). Therefore, the FITC was conjugated to N- terminal of the peptides. To study whether the peptide really binds to FRα, we employed RNA interference to specifically knockdown FRα expression in SKOV3 cells. We observed that the binding of C7 peptide was decreased significantly after FRα silencing. This experiment confirmed that C7 peptide binds to SKOV3 cells through FRα. In this study we aimed to discover a tumor targeting peptide and the FITC conjugated peptide was used to evaluate the tumor-targeting ability *in vivo* and the result showed that except for the liver and kidney, C7 was accumulated at the site of tumor tissue, indicating that the peptide has the ability to target tumor tissue without phage environment. The FITC conjugates peptide can also be internalized into cells. The result is encouraging as it suggests that the peptide might be an appropriate carrier not only for targeting tumor cells but also for delivery of cytotoxic drugs into the cells. Further investigations in the future are planned to define the internalization mechanism of the peptides.

By computational docking, we analyzed the interaction between the peptide and FRα. In this computer model, the peptide binds at the entrance of the folate binding pocket, but does not stick into the pocket. C7 interacts with Tyr60, Arg61, Ser101, Lys19, Trp102, Lys136, Gly137 and Trp140 of FRα. Some of these amino acids (Tyr60, Trp102, Lys136, Gly137, and Trp140) also interact with the glutamate group of folate at the entrance of the folate-binding pocket. However, these amino acids do not play important role in binding with folate because mutations of these amino acids did not abolish the expression level of FRα or decrease the affinity between FRα and folate^19^. Typ171 and Asp81, which locate inside the folate binding pocket, interact with the pterin ring of folate and are considered as key contributors to high-affinity folate binding^19^. However, they do not interact with the peptide C7 on our binding model. The peptide, because of the size, cannot dock into the folate binding pocket but binds to the surface of the receptor. This different binding mode may explain its relatively weak binding to FRα with a KD value of 0.3 μM. In general, the affinity of peptide is lower compared to antibodies. Different strategies can be employed to improve the affinity of C7. One strategy is to change the stereochemistry of a peptide. For example, a D-amino acid peptide ligand showed a 10-fold improvement in affinity compared to its L-amino acid counterpart^25^. Another strategy is dimerization or trimerization of a peptide. After dimerization, the affinity a PSMA-specific peptide was dramatically enhanced^26^. In our future studies, we will begin with dimerization to improve the binding affinity of C7.

Most residues involved in folate binding are identical among different subtypes, indicating that the folate binding interactions are probably conserved in all three different folate receptor subtypes. Unlike anti-FRα antibodies, folate conjugates target both FRα and the functional form of FRβ and this might reduce the selectivity for tumor cells. It is interesting to be indicated by computational docking that the peptide C7 form hydrogen bond with R61, which is a unique amino acid at the entrance of folate binding pocket in FRα but not present in other folate receptor subtypes (Supplementary Fig. S1). This unique binding give rise to the hypothesis that the peptide C7 may target FRα specifically without affecting FRβ, avoiding the possible adverse effects of the folate conjugates. Further experimental works are still needed to prove this hypothesis.

## Conclusion

In conclusion, we have discovered and characterized C7 as a potent and selective peptide ligand for FRα. C7 showed specific binding to FRα expression cells and tumor targeting ability *in vivo*. This peptide can be internalized into SKOV3 cells and the affinity for FRα is around 0.3 μM. As a tumor-specific peptide ligand, C7 has great potential for delivery of cancer therapeutics or imaging agents to FRα expressing tumors.

## Materials and Methods

### Library Screening

Recombinant folate receptor α was expressed in *E. coli* system and purified with a Ni-column. The purified recombinant protein was dissolved in carbonate buffer (pH 9.6) and coated on polystyrene 96-well microtiter plates (Corning, Inc., NY, USA) overnight at 4 °C at a concentration of 50 μg/mL, 100 μL per well. The next day, each well was blocked with 200 μL 3% (w/v) bovine serum albumin (Sangon Biotech Co., Ltd., Shanghai) dissolved in 0.01 M TBS (pH 7.4) for 2 hours at 37 °C. After washing 3 times with TBS containing 0.05% (v/v) Tween 20 (TBST), 100 μL Ph.D.-12 peptide library aliquot (New England Biolabs, Inc., USA) containing 10^11^ phages was added to each well and the plate was incubated for 1 hour at 37 °C. After washing 10 times with TBST and 3 times with TBS to remove the unbound phages, the target-bound phages were eluted with 150 μL 100 mM Glycine-HCl (pH 2.2) and neutralized immediately with 9 μL 2 M Tris-HCl (pH 9.1). 5 μL of eluate was used for titering and the rest was used to infect *E. coli* ER2738 host strain (New England Biolabs, Inc., USA) for amplification. Phage particles were rescued from the cells and used for the subsequent round of target selection. The rescue-selection-plating cycle was repeated 4 times with reduced concentration of coating protein. Polyclonal phage ELISA was performed to evaluate the enrichment of FRα binding phage after each round screening. 94 individual clones were analyzed for specific antigen binding by phage ELISA. Phage titer and amplification was performed as previously described^24^. Phage recovery rate of each round was calculated as: titer of output phage/titer of input phage × 100%.

### Phage ELISA Assay

96-well microtiter plates were coated with 100 µL 10 µg/mL recombinant FRα in 0.05 M sodium bicarbonate (pH 9.6) and the plate was kept overnight at 4 °C. After washing the plate three times with TBST, the plate was blocked with 3% BSA at 37 °C for 2 h. For polyclonal phage ELISA, after each round of screening, the phage eluate was amplified and 100 μL 10^10^ phages diluents were added to each well and incubated for 2 h. To analysis individual phage clones, each phage clone was amplified and 10^10^ phages were added to each well (100 µL /well) and incubated for 2 hours at 37 °C. After washing the plate for 3 times with TBST and 3 times with TBS, 100 µL of HRP-conjugated anti-M13 antibody (1:10000, Sino Biological, Inc., Beijing) was added and the plate was incubated for 1 hour at 37 °C. After washing, the tetramethylbenzidine (TMB) (Sangon Biotech Co., Ltd., Shanghai) substrate (100 μL/well) was added to the wells and the reaction lasted for 15 minutes. The reactions were stopped with 2 M sulfuric acid (50 μL/well). The absorbance of each well at 450 nm was detected with an automated ELISA reader.

### Cell-based ELISA

Phage clones with high ELISA value were further tested by cell-based ELISA. SKOV3 (human epithelial ovarian cancer cell) is a FRα expressing cell line and was used to evaluate the binding of selected phage clones to natural FRα protein on cell surface. HEPG2 (Human Liver Carcinoma Cell) without FRα expressing was used as a negative control. Both cell lines were purchased from Shanghai Cell Biology Institutes (Academia Sinica, Shanghai, China) and maintained in RPMI 1640 Medium with 10% fetal bovine serum and antibiotics. The presence or absence of FRα expression of the two cell lines were confirmed by flow cytometry described in 2.4. The cells were cultured in 96-well plate to 80% confluence and fixed with 4% paraformaldehyde. After blocking with 3% BSA for 2 h, 10^10^ individual phages were added to each well and incubated at 37 °C for 2 h. Then the plate was washed with PBST for three times and cell-bound phage was detected with HRP-conjugated anti-M13 IgG (1:10000) as described in phage ELISA.

### Flow Cytometry

SKOV3 and HEPG2 were tested individually for their expression of FRα. The cells were collected and suspended in 2% FCS-PBS medium at 1 × 10^6^ cells/mL and incubated with polyclonal anti-FRα antibody (1:1000, Sino Biological, Inc., Beijing) at 4 °C for 1 h. After washing the cells twice with 2% FCS-PBS by 5 min centrifugation at 300 g, the cells were stained for 30 min at 4 °C with AF647 conjugated goat anti-mouse antibody (Fcmacs, Inc., Nanjing). Cells were subjected to fluorescence analysis using a flow cytometry analyzer (Guava® easyCyte 6-2 L, MilliporeSigma, USA).

SKOV3 cells were collected and suspended in 2% FCS-PBS medium at 1 × 10^6^ cells/mL. Purified phage clones were incubated individually with the cells at 4 °C for 1 h. The cells were washed twice with 2% FCS-PBS by 5 min centrifugation at 300 g. Mouse anti-M13 antibody was diluted with 2% FCS-PBS (1:400) and incubated with cells for 30 min at 4 °C. After washing, the cells were stained for 30 min at 4 °C with FITC conjugated goat anti-mouse antibody (Fcmacs, Inc., Nanjing). Cells were analyzed as described above.

### FRα knockdown by RNA interference

FRα targeting siRNA (sense: GGACUGAGCUUCUCAAUGUTT, antisense: ACAUUGAGAAGCUCAGUCCTT) and the control siRNA (sense: UUCUCCGAACGUGUCACGUTT, antisense: ACGUGACACGUUCGGAGAATT) were designed and synthesized by Shanghai Genepharma RNAi company. SKOV3 cells were cultured in 6-well plate to 70% confluence and the siRNA were transfected into SKOV3 cells using SuperFectin siRNA Transfection Reagent (Pufei Biological, Inc., ShangHai). After 72 h, siRNA mediated silencing of FRα was tested with monoclonal anti-FRα antibody (1:1000, Sino Biological, Inc., Beijing) using flow cytometry and the binding of C7 peptide to SKOV3 with or without RNA interference was analyzed.

### *In Vivo* Homing Study of Phage Clones

Female Balb/c nude mice aged 6–8 weeks were purchased from the Yangzhou University Animal Center. All the animal studies were performed in compliance with the National Institutes of Health Guide for the Care and Use of Laboratory Animals and approved by IACUC (Institutional Animal Care and Use Committee of China Pharmaceutical University). The authors confirm that experiments involving laboratory animals adhered to the ethical standards of National Institutes of Health Guide and China Pharmaceutical University. The laboratory animals were used under the experimental animal production license 2121922. All animals were housed in a controlled environment (25 °C; 12 h light-dark cycle), with water and food provided freely. 2 × 10^6^ SKOV3 cells were subcutaneously injected into the left flank of mice to generate epithelial ovarian cancer xenografts. 2 × 10^11^ individual purified phage clones were injected intravenously and circulated for 15 min. After anesthetized with 10 mg/kg chloroquine (5% w/v), the mice were perfused through the heart. Then, the tumor and main organs (liver, lung, kidney, heart and ovary) were harvested, weighed and homogenated. The homogenated tissues were washed, and lysed with 1% NP40. Bound phages were recovered by elution and titrated as described^24^. The distribution of phages was calculated as phages per gram.

### Tumor Targeting of Conjugated Synthetic Peptides

FITC conjugated peptide C7 was chemically synthesized by GL Biochem (Shanghai, China) and purified by high-performance liquid chromatography with more than 95% purity and confirmed by mass spectrometry. The FITC conjugated peptide was evaluated in tumor bearing mice for its tumor targeting capability. The peptide was dissolved in PBS at a concentration of 10 mg/mL and 200 μL was injected intravenously into a tumor bearing nude mouse. After 2 h circulation, tumor and other organ tissues (liver, lung, kidney, heart, and ovary) were harvested and analyzed using Carestream IS4000 fluorescence imaging system.

### Affinity test using Microscale Thermophoresis (MST)

FITC conjugated peptide C7 was diluted with PBS, pH 7.4. Serial dilutions of peptide were mixed with purified FRα protein (1 μg/mL) and incubated for 5 min at room temperature. Each sample was loaded into the standard monolith NT capillary and detected in Monolith NT.115. According to the changes of fluorescent thermophoresis signal caused by the serial concentrations, the equilibrium dissociation constant (KD) value was calculated from Hill formula by the NT analysis software.

### Cell internalization of Synthesized Peptide C7

To visualize internalization of synthesized peptide C7 into cells, SKOV3 or HepG2 cells were seeded in 24 well plates (10^5^ cells/well) containing coverslips and incubated for 24 hours in RPMI 1640 medium with 10% FBS. FITC conjugated peptide C7 or PB-TUP^24^ (VHWDFRQWWQPS) (200 μg/mL) was incubated with the cells for 4 hours at 37 °C. After incubation, the cells were washed once with PBS and fixed in 4% paraformaldehyde for 10 min. Cells were then washed three times with PBS and stained with DAPI for 20 min at room temperature. Internalized fluorescent signals were imaged with a Zeiss LSM800 confocal microscope.

### Circular Dichroism Spectroscopy

Circular dichroism (CD) spectroscopy was performed on a Jasco J-810 spectropolarimeter using a 1-mm cuvette. The peptide was dissolved in ultrapure water at a concentration of 0.3 mg/mL. Spectra were collected every 1 nm from 260 to 190 nm. The CD spectra are reported as CD (mdeg).

### Molecular Docking and Molecular Dynamics Stimulation

The 3D structures of C7 peptide were predicted by Pepfold3, a peptide structure prediction server. It predicts the structure of peptide based on the Hidden Markov model conformation sampling approach^27^. The peptide sequence was imported into the workspace of Pepfold3 and allowed to run. Predicted 3D structure was noted in PDB format. The crystal structure of folate receptor α was obtained from the Protein Data Bank (www.rcsb.org) (PDB ID: 4LRH), and then the ligand folate was extracted as a docking receptor. Interaction between peptide and folate receptor α was analyzed by applying ClusPro protein-protein molecular docking programme^28–31^. ClusPro’s docking algorithms calculated the billions of values for putative complexes and determined a reset number with favorable surface complementarities^30,32^. In the docking analysis, top-docked complex was detected and assessed by molecular dynamics (MD) simulation in order to refine and validate the protein interface. MD simulation was performed using the GROMACS package v.4.5.5. The interface of interactions was analyzed with MOE 2014.09. The high-quality images of molecular models of the complexes were rendered using Pymol.

### Statistical analysis

All statistical analyses were performed by using GraphPad Prism 6.0 software (GraphPad software Inc., San Diego, CA, USA). Unpaired two-tailed student’s t-test was used to analyze the differences between means. All data are expressed as the mean ± SD. The error bars represent the standard deviation of three independent determinations. Asterisks indicate significant level versus the control condition: *P < 0.05, **P < 0.01, ***P < 0.001.

## Electronic supplementary material

supplementary information
