# Supplementary material for: Identification of a peptide for folate receptor alpha by phage display and its tumor targeting activity in ovary cancer xenograft
Source: Sci Rep. 2018 May 30;8:8426. doi: 10.1038/s41598-018-26683-z (PMC5976665; doi:10.1038/s41598-018-26683-z)
Supplement: Supplementary file 1 — supplementary information [file 41598_2018_26683_MOESM1_ESM.docx]

**Identification of a peptide for folate receptor alpha by phage display and its tumor targeting activity in ovary cancer xenograft**

Lijun Xing^1^, Yifeng Xu^1^, Keyong Sun^1^, Hong Wang^1^, Fengguo Zhang^1^, Zhengpin Zhou^1^, Juan Zhang^1^, Fang Zhang^2^, Bilgen Caliskan^1^, Zheng Qiu^1^* & Min Wang^1^*

1. School of Life Science and Technology, China Pharmaceutical University, Nanjing, 210009, P.R. China

2. Jiangsu Collaborative Innovation Center of Chinese Medicinal Resources Industrialization, School of Pharmacy, Nanjing University of Chinese Medicine, Nanjing, 210023, P.R. China

*Correspondence to Prof. Min Wang and Prof. Zheng Qiu

School of Life Science and Technology, China Pharmaceutical University, Nanjing, 210009, P. R. China. Tel: 86-25-83271395; Fax: 86-25-83271007;

E-mail: minwang@cpu.edu.cn(M Wang)

qiuzheng@cpu.edu.cn(Z Qiu)


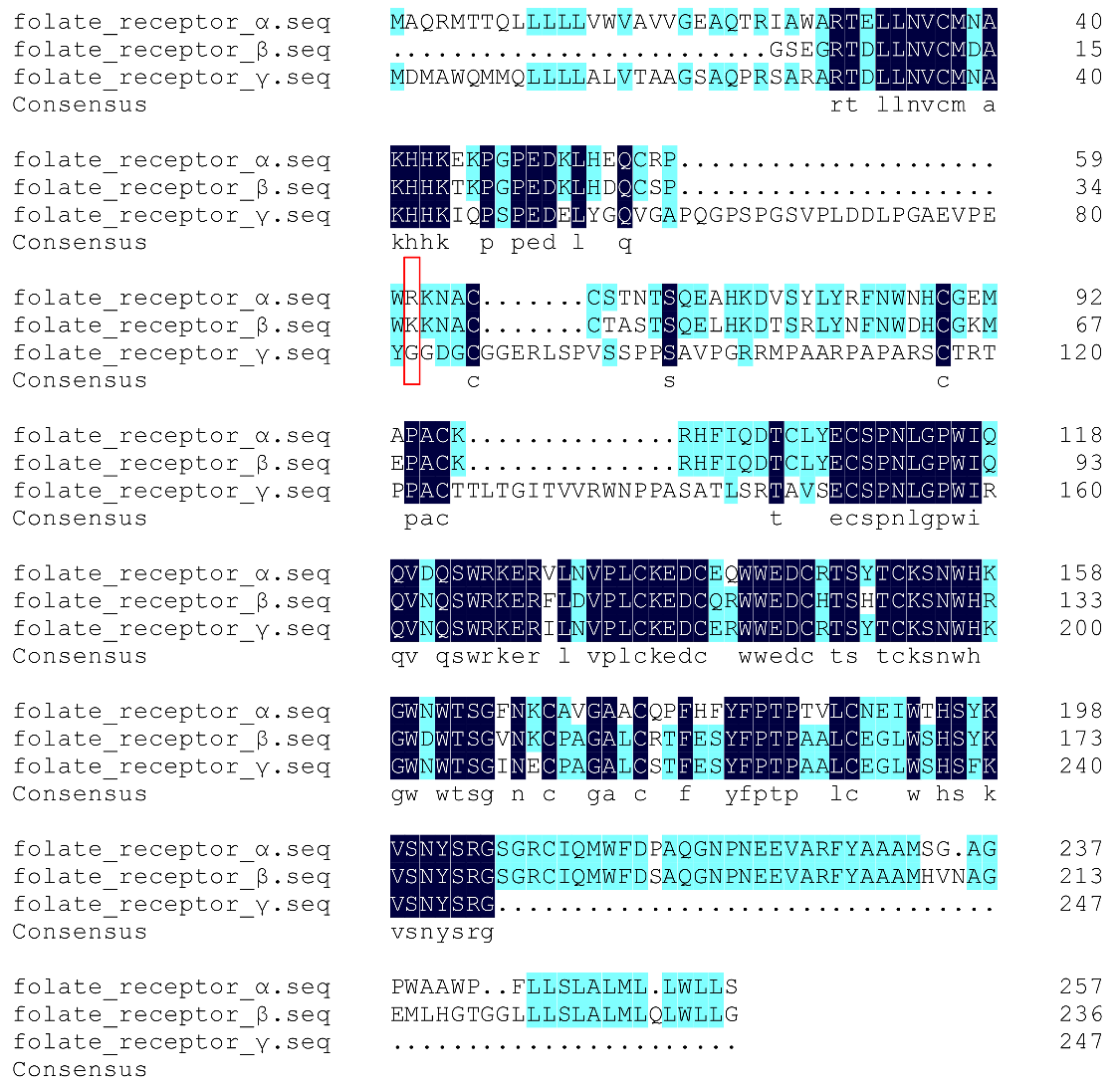


**Supplementary Figure S1.Sequence alignment of human folate receptor α, β, γ subtypes.** The unique amino acid R61 in FRα is indicated by a red box.
